# The impact of the COVID-19 pandemic on mental health and quality of life in people living with and beyond breast, prostate and colorectal cancer – a qualitative study

**DOI:** 10.1186/s40359-023-01471-1

**Published:** 2024-01-16

**Authors:** Simon Pini, Caroline Buck, Phillippa Lally, Rebecca Beeken, Abigail Fisher

**Affiliations:** 1 Leeds Institute of Health Sciences, University of Leeds, Level 10 Worsley Building, Clarendon Way, Leeds, LS2 9NL UK; 2 UCL Department of Behavioural Science and Health, University College London, 1-19 Torrington Place, London, WC1E 7HB UK; 3 Department of Psychological Sciences, University of Surrey, Guildford, Surrey GU2 7XH UK

**Keywords:** Cancer survivorship, Mental health, Quality of life, COVID-19, Pandemic, Qualitative, Needs

## Abstract

**Background:**

Individuals living with and beyond cancer are at heightened risk of adverse psychological and social outcomes and experiences. In March 2020, the COVID-19 global pandemic presented a unique set of social circumstances with the potential to exacerbate the challenges faced by this population. The purpose of this study was to investigate the experiences of people living with and beyond cancer during the first year of the COVID-19 pandemic and assess the impact on psychological and social aspects of their lives.

**Methods:**

From a pool of participants from a larger health behaviours study thirty participants were purposively sampled for characteristics including: diagnostic group (breast, prostate and colorectal cancers), gender, time since diagnosis and age. Semi-structured interviews were conducted via telephone to discuss their experience of living through the pandemic. A thematic analysis was conducted using a needs-based approach to detail the fundamental needs expressed by this population in relation to their mental health and quality of life during the pandemic.

**Results:**

Three fundamental needs underpinned the experiences expressed by participants: *the need to feel safe;* particularly in relation to risk of contracting COVID-19 and their ongoing cancer monitoring; *the need to feel connected;* to the people, places, activities and practices of everyday life; and *the need to make the most out of life*; specifically in context of having already endured cancer and cancer treatment. Participant experiences are described in relation to how they impacted each of these three needs.

**Conclusions:**

People living with and beyond cancer have past and ongoing experiences that make them vulnerable to adverse psychosocial reactions and outcomes. Support for this population needs to provide greater clarity of risk, clearer guidelines specific to their personal circumstances, and regular updates on scheduling of important follow up care and monitoring.

**Supplementary Information:**

The online version contains supplementary material available at 10.1186/s40359-023-01471-1.

## Introduction

In March 2020 the World Health Organisation (WHO) declared the COVID-19 outbreak a global pandemic, leading to a public health emergency affecting the well-being of individuals and communities across the world [1]. Multiple stressors contributed to heightened incidence of anxiety and depression during this time, including; limited access to medical care, severe shortages of resources, economic uncertainty and concerns about contracting the virus [2, 3]. Furthermore, significant social restrictions and diminished contact with family, friends and colleagues has resulted in feelings of social isolation and loneliness [4]. These findings align with previous research conducted during health emergencies such as the 2009 SARS outbreak, which identified various psychiatric morbidities experienced by the general population in the face of health crises [5, 6].

Even without the amplifying impact of a global pandemic, individuals living with long-term health conditions such as cancer have a heightened risk of developing psychosocial disorders such as anxiety and depression [7, 8]. A broad range of biological and individual factors are thought to play a critical role in the development of these disorders among people living with and beyond cancer (LWBC), including disease and treatment characteristics, prognosis, personality type, socioeconomic status, social support and family history [9]. Many individuals risk chronic morbidity and premature mortality as a consequence of their disease [10], with cancer and treatment-related sequelae like fatigue, sleep disturbances, pain and fear of cancer recurrence often persisting after treatment completion and affecting mental health and quality of life [11–15].

It is feasible therefore that the COVID-19 pandemic might present greater challenges to people LWBC than those without a long-term health condition [16]. This was previously demonstrated during the Sars-Cov-2 virus, which resulted in higher rates of intensive care admission and mortality in people LWBC compared to those without a cancer diagnosis [17–19]. Heightened risk of severe disease from COVID-19 has also been shown in those whose cancer is in remission and no longer receiving active cancer treatment [18]. Such evidence prompted the NHS to advise 2 million clinically vulnerable people (including some people LWBC) in the United Kingdom to stay at home and avoid social contact in the early stages of the pandemic [20]; subsequent quantitative data has shown that social restrictions contributed to increased stress, depression and anxiety among people with chronic conditions compared to those without [21]. A prospective, multicentre cohort study of 1051 women with breast cancer showed a deterioration of psychosocial wellbeing as a result of lockdown measures, with half of respondents experiencing severe loneliness [22]. Numerous other quantitative studies have explored the impact of lockdown on delays to accessing cancer care and interruptions to treatment on patients currently waiting for or receiving cancer treatment, finding similarly high levels of fear of disease progression, anxiety, and depression (e.g. [23, 24]).

Whilst investigations into physical symptoms and treatment side effects in this population are widespread, in-depth explorations of their mental health tend to get less attention [25]. The limited research concerning the impact of the pandemic on the mental wellbeing of people LWBC has shown that many felt a heightened threat of COVID-19, feelings of vulnerability and fear over treatment disruptions [26], were fearful about the consequences of isolation and lockdown on their mental health [27], had concerns about changes to family dynamics, and experienced feelings of boredom due to limited social interactions [28]. Few studies examine these issues in-depth using qualitative methods, missing the opportunity to learn lessons that could contribute to preparedness and improved responses in future health emergencies.

The aim of the current study was therefore to qualitatively explore the impact of the COVID-19 pandemic on the wellbeing of men and women living with and beyond breast, colorectal and prostate cancer in the first year of the pandemic in the United Kingdom.

## Methods

### Design

This qualitative study was underpinned by a constructivist epistemology, and used a single time-point telephone interview to explore how people LWBC made sense of the impact of the COVID-19 pandemic on their mental health and wellbeing.

### Participants

Participants were sampled from a randomised controlled trial of a habit-based health behaviour intervention: Advancing Survivorship After Cancer: Outcomes Trial (ASCOT) [29]. ASCOT participants included adults (aged ≥ 18 years), with a self-reported diagnosis of non-metastatic breast, prostate or colorectal cancer, who were not receiving active anti-cancer treatment (except for oral anti-cancer treatments taken at home), and were able to understand spoken and written English. Recruitment to ASCOT was conducted between 2015 and 2019.

During an ASCOT survey in 2020, participants indicated if they would be interested in an additional interview focussing on their experience of COVID-19. This resulted in a pool of 620 interested participants, who were subsequently purposively sampled for the qualitative study based on demographic characteristics (age, gender, diagnosis, geographical location, marital status, ethnicity and ASCOT experimental group allocation). Ethical approval for the COVID-19 follow-up was granted by South Central – Oxford Ethics Committee in September 2020 as an amendment to the original ethical approval for ASCOT.

### Data collection

One-to-one telephone interviews were conducted with selected participants. A semi-structured interview schedule (see Additional file 1) used open questions to explore their experience of the COVID-19 pandemic. Interviews were conducted by SP and CB who are both experienced qualitative researchers with no prior relationship with participants. Interviews were guided by the participants wherever possible in order to pursue experiences related to mental health and quality of life for detailed and reflective discussion. Interviews were audio recorded and transcribed verbatim for analysis, with identifiable information removed.

### Analysis

Analysis was guided by a flexible thematic approach, as recommended by Braun and Clarke [30, 31]. Six stages were followed: 1) familiarisation; 2) coding; 3) searching for themes; 4) reviewing themes; 5) defining and naming themes; 6) writing up. Analysis was led at all stages by the first authors (SP and CB). Each stage was supported through discussion of emerging ideas with the other authors. During the 4^th^ and 5^th^ stages of the analysis, the authors adopted a needs-based approach [32] to facilitate the definition of the themes. During this stage the authors continually reflected on the question: “What need is being impacted here?”. This approach helped move the analysis away from categorical labels and towards the more fundamental needs that were being affected by the pandemic for people LWBC.

## Results

### Participants

From the 620 potential participants, an initial 10% shortlist (*n* = 62) was created based on the sampling characteristics. Thirty-two participants were approached in the first instance to represent diversity of cancer diagnoses, gender, age, ASCOT experimental group allocation and living conditions. Two (6%) declined to participate, explaining their reasons as current physical illness and depression. Ten participants from each cancer diagnosis cohort were interviewed, and sample characteristics are shown in Table 1. The most prevalent characteristics were white-British (75%), married (70%) and aged 50–70 (77%). Living location was self-reported from a list of options within the ASCOT surveys.

**Table 1:** Participant characteristics

	N	%
Total	30	100
**Gender**		
Female	16	53
Male	14	47
**Age-group**		
39–49	4	13
50–59	11	37
60–69	12	40
70 +	3	10
**Diagnosis**		
Breast	10	33
Prostate	10	33
Colorectal	10	33
**Marital status**		
Married/living with partner	21	70
Single	6	20
Divorced	3	10
**Living location**		
City	12	40
Village	9	30
Town	7	21
Hamlet or isolated dwelling	2	6
**Ethnicity**		
White British	22	75
White other	2	6
Black Caribbean	3	10
Black African	2	6
Asian	1	3
**Time since most recent diagnosis (years)**		
Under 6.5	4	13
6.5 – 7.5	11	37
7.5—8.5	10	33
Over 8.5	5	17

Interviews were conducted between November 2020 and February 2021 and lasted 35–90 min. To provide context for the results reported in the subsequent sections, an overview of the cycles and stages of lockdown in the United Kingdom is provided in Table 2.

**Table 2:** Cycles of COVID-19 lockdown (March 2020-February 2021)

**March**	The United Kingdom goes into the first national lockdown.
**April**	Lockdown is extended in combination with a set of recommended targets for the government to meet before it is lifted.
**May**	Plan for conditional easing of lockdown, including return to work for those who cannot work from home.
**June**	Phased reopening of schools and non-essential shops. Easing of two-metre social distancing rules, but then implementation of local lockdowns by the end of the month.
**July**	Local lockdowns applied to more areas, with others allowed to reopen certain businesses (pubs, restaurants, hairdressers). Local authorities given more powers to make local decisions.
**August**	Further easing of lockdowns with encouragement for the public to go back out to restaurants /other leisure facilities.
**September**	“Rule of six” introduced for social gatherings. Return to working from home and a 10 pm curfew introduced.
**October**	Introduction of the “tier” system for local lockdowns, followed by the second full national lockdown.
**November**	Second national lockdown in force
**December**	Many places given highest “tier 4” status, but restrictions relaxed for the Christmas period to allow minimal gatherings.
**January**	Third full national lockdown.
**February**	Publication of “roadmap” to systematically lift restrictions across the United Kingdom.

A clear summary of the tier system can be found here: https://www.ageuk.org.uk/information-advice/health-wellbeing/conditions-illnesses/coronavirus-guidance/local-lockdown-tiers/

### The impact of the COVID-19 pandemic on the needs of people LWBC

A thorough analysis of the transcripts resulted in the identification of three fundamental needs that were being impacted by the challenges of the COVID-19 pandemic for those LWBC:

- *I need to feel safe*: how participants experienced their risk to COVID-19 and the impact on their usual cancer follow up.
- *I need to feel connected*: the impact of social restrictions on participants’ relationships with people and places around them.
- *I need to make the most out of my life*: balancing pandemic related restrictions with their desire to make the most out of their life having successfully completed cancer treatment.

The impact of the pandemic on these needs was raised and discussed by participants across the sample, and are described in the following sections.

### I need to feel safe

The need to feel safe was described by all participants and focussed on two main areas. For many there was a primary focus on their perceived risk of contracting COVID-19, alongside a secondary safety need related to their cancer diagnosis, ongoing follow-up, and any long term symptoms/side-effects. This secondary need had often been brought into sharper focus because of the impact of the pandemic on reliable and regular follow up routines.

Despite only a minority of participants reporting having contracted COVID-19 by the time of their interview, all discussed the degree to which they felt vulnerable during the pandemic. A number had received letters advising them to shield because of their cancer diagnosis and comorbidities. Whilst most participants broadly trusted the expert advice, some described guidelines as “*confusing”* and said they were weighing the evidence and making their own decisions. Several participants acknowledged they felt vulnerable to COVID-19 and infections in general, but also that the consequences would be worse for them as people LWBC than for the general population.

A number of participants reported they had “*done my five years”* and therefore their original cancer diagnosis left them feeling no more susceptible than anyone without cancer and seemed surprised that others thought so. Several participants discussed managing the heightened concerns that family, friends and employers had for their vulnerability, which was sometimes misaligned with their own. This could be challenging for participants who were unsure how to balance acknowledging these concerns, being sensitive to the worries of loved-ones, but also trying to freely live their life.

“I think they do tend to be protective, I mean I would probably say overly protective because I tend to more be, like I throw caution to the wind I think and have a life, but I care very much about them and the last thing I want to do is worry them more than they were already worried” (Male, prostate cancer, 72)

Others who felt more vulnerable discussed the precautions they were taking to protect themselves and those around them. These participants were often older, had comorbidities, or had partners or family members with their own health conditions, and therefore had a sense of personal vulnerability or responsibility for others. However, it seemed all participants were following recommended social distancing and wearing masks as required. A small minority extended PPE to gloves, visors and higher quality masks. Many reduced the frequency of their trips to busy areas, such as supermarkets, and remained in familiar places they knew would be quiet at certain times. A small minority took this further and isolated at home at all times. For some this was fuelled by a distrust of other peoples’ appropriate adherence to guidelines, as well as a genuine fear of being vulnerable in these situations.

“it initially had quite a dramatic impact psychologically, I used to be frightened to go to work” (Female, breast cancer, 58)

“I had a bus journey which I think was to go for my colonoscopy, there were people who literally had their masks round their chins and they were talking on their mobile phones on the bus, and it makes you quite wary, it makes you quite frightened but there’s nothing that you can say”(Male, colorectal cancer, 69)

During the pandemic the reliable follow-up and maintenance routines of participants were disrupted. Participants reported being scheduled to have tests, scans, check-ups and procedures related to their cancer follow-up and monitoring. These were frequently postponed or reconfigured as telephone or virtual appointments, which were often more convenient for participants, but lacked the same level of reassurance as face-to-face contact with their familiar care team.

“I think there’s something about seeing somebody that makes it more reassuring than if you speak to somebody on the phone”(Male, colorectal cancer, 59)

Despite often being in longer-term follow up, it was clear that for many participants the uncertainty presented by the pandemic highlighted how important these periodic appointments remained for their perception of safety and stability, described by one as their ‘*security blanket*”. Many discussed needing the comfort of receiving reassuring test results, and often feeling anxious prior to appointments, being hyper-vigilant about possible symptoms. Uncertainty surrounding rescheduling tests or missing them altogether often increased or prolonged these feelings of anxiety. Even without potential symptoms, participants remained concerned about the possibility of cancer recurrence and relied on these monitoring appointments to provide them with reassurance and a stable footing to move forward with confidence. Anxiety for participants during the pandemic was often fuelled by news stories chronicling the severe delays in cancer care and diagnosis, as well as the barriers to accessing General Practitioners.

Many participants were also having to cope with comparable situations relating to comorbidities. This amplified anxieties and uncertainty, as well as the administration related to organising and chasing rescheduled appointments. Participants who felt confident and had a sense of agency were able to navigate the healthcare system to assess the situation regarding follow up appointments, but others felt more helpless and were left wondering when and if appointments would ever be re-scheduled. Media portrayal of overrun hospitals made some participants feel they would be unsafe in that environment or unnecessarily bothering busy healthcare staff with their enquiries.

“for the moment I let it go because the health service is busy, but when the situation normalises I think I will call the hospital and say, “Is it an accident or is it meant to be like this, is it meant to be like this or is it an oversight?”(Male, prostate cancer, 65)

There were some participants who were still required to attend hospital for more urgent oncology procedures, which was a distressing and worrying experience in the uncertainty of the pandemic and the infection possibilities. Some participants described being “*frightened to go to the hospital”* and finding the PPE and infection control procedures reassuring but also visually unsettling.

“the most frightening one was when I went to hospital and it was on the news….the staff were both sides of the corridor clapping and chatting, not having masks on and they were releasing one of their staff members who had been confined for a few months with COVID…so they were all happy, but I had to walk the gauntlet to get to the area for my MRI. I was quite concerned.” (Female, colorectal cancer, 58)

Many participants noted that the NHS appeared under resourced/funded prior to the pandemic, so the onset of COVID-19 served to heighten this unease, with many expressing grave concerns that the pandemic would expose the vulnerability of the NHS even further. The need for safe and reliable monitoring for people LWBC meant they felt this vulnerability acutely.

### I need to feel connected

Unsurprisingly, the effects of enforced social restrictions was felt strongly by participants and significantly impacted their connection to the important people and places around them. The lack of access to family and friends was cited frequently as representing the worst elements of the pandemic.

Participants who felt that their cancer diagnosis made them no more vulnerable to COVID than anyone else appeared particularly needful of connection and family closeness. This seemed to be because their diagnosis was no longer integral to their self-concept and therefore additional restrictions felt unfair. Others who still thought of themselves as being ‘someone who had cancer’, appeared to be more accepting of the compromises in connection with others during this time.

Whilst some participants described an increasing reliance on technology (e.g., Zoom) to stay in touch with friends and family, and to access online physical activity classes such as yoga/Pilates, for many (especially older participants) restriction to the everyday aspects of day-to-day living (a casual coffee with a friend, a hug from a grandchild, a trip to the shops) seemed to have a greater effect than the loss of more monumental markers of freedom (trips abroad, parties etc.). This loss of liberty inevitably raised philosophical questions as to the meaning of life, citizenship and their place in the world. For many, the impact of these restrictions were felt more acutely because of the previous restrictions they had experienced related to their cancer and treatment.

“I’m not worried about going on holiday, but to have people in your house...in the garden...I think we’ve realised what’s important to each of us … it’s the bond that puts it all together...you just want to be with them (people), you want to share....it’s experiences, isn’t it?” (Female, breast cancer, 65)

However, the impact of strict social restrictions appeared to differ strongly according to baseline/pre-pandemic attitudes to friends, social activity and need for company. Many expressed gratitude for the opportunity of having people around them; the importance of having company and support was deemed critical to getting through difficult days and seemed to compensate for the absence of friends and wider family. Clearly this appreciation didn’t preclude fraying nerves and feelings of claustrophobia, but many viewed the enforced family dynamics as helping to maintain morale. Several participants reported that having family around them was a way of avoiding introspection and loneliness, recognising that inactivity could lead to sadness and indolence. Others found the restrictions led to renewed spousal relationships and understanding, and a time of change and reflection. The presence of family—albeit challenging/claustrophobic—was seen as being better than being alone. The impact on relationships was also exacerbated by previous cancer experiences. Cancer diagnosis and treatment inevitably tested relationships in much the same way as the pandemic, by strengthening some relationships through support and understanding, but straining others through the difficulties of coping with the cancer experience.

“I feel fortunate in some ways….we all get on well together…I feel that although I’m missing out on all my friends, I have a good balance …and that for me fills a hole that I think I would feel if it were just my husband and I” (Female, Breast cancer, 55)

However, other participants admitted lockdown served to emphasize relationship difficulties that were present pre-pandemic; that people can “*feel lonely even surrounded by others*” was mentioned by a number of participants, who found enforced inseparability very challenging. Several participants described quite significant feelings of depression brought about by the imposed pause in their usual activities and subsequent reflection on the quality of relationships in their lives. This was often amplified by a feeling that they should be doing more, or deserve more, because of surviving their cancer experiences.

Some participants recalled how the return of their adult children to the family home served to readdress the faults of the past and forge stronger family bonds. Lockdown was perceived by several as an unexpected gift that gave them the opportunity to create new memories and augment the family union, not least because there was almost no choice but to be together, eat together, talk more and share common experiences.

“I was able to spend more time with my husband which I had never really been able to do before, the children were at home, and we haven’t really had that kind of good quality family time in, I think really I’d say we’ve never really had it at home”(Female, colorectal cancer, 39)

Several participants described difficulties with grief for loved-ones or friends who had died during the pandemic and how hard it was to not be able to attend funerals or comfort those around them. Restrictions to shared experiences and rituals associated with death made it more difficult for participants to grieve in the way they wanted to.

“it’s been roller coasting with losing friends and other people….I went to the funeral for all of them…they got two children, and I have to support the children with the funeral and everything. I have to support them, because they haven’t got anybody” (Male, prostate cancer, 62)

### I need to make the most out of my life

Several participants expressed the view that they had already coped with cancer and could therefore ride out whatever COVID-19 presented to them. This seemed more prevalent in those whose cancer diagnosis was further behind them and were living with fewer/less severe long-term effects.

“I think it’s a fatalist view isn’t it, if the last year of my life was Covid and the restrictions, what an end to a life. Life is too short for all this to be affecting us” (Male, prostate cancer, 80)

However, a number of older participants expressed a strong sense of sadness about time passing during the pandemic, with uncertainty about how to balance risks with the desire to still make the most out of their life. Some discussed frustration at “*losing this year”* having already lost time to cancer treatment, and the regret of not being able to make the most of their life living beyond cancer. This inevitably raised yet more philosophical questions about life’s meaning, their choices and what might lay ahead.

“I just want to enjoy what bit of life you might have left rather than sitting at home indoors and not being able to go out”(Male, prostate cancer, 78)

“We can’t all put everything on hold forever….I’m getting too old, I don’t want to be too old to do the things I want to do, I want to travel, I want to do nice holidays and see places I haven’t seen while I’m physically able to do so” (Female, colorectal cancer, 76)

Whilst some participants reported the restrictions posed by the pandemic were less impactful compared to their cancer diagnosis, the reduction in freedom and ‘stay at home’ directives were felt particularly keenly by participants who had comorbidities and continuing treatment/ill health; a specific and palpable sense that *‘time was running out’* for them and a fear that a particular milestone (birthday, Christmas) *‘might be my last’* punctuated their lives. Some participants recalled how their cancer diagnosis, whilst challenging, was experienced in ‘normal’ times with familiar rituals and comforts still enjoyed, but that the pandemic had taken this important mechanism away.

“I feel resentful... there’s probably people far worse off than me … (but) you want to enjoy what bit of life you might have left rather than sitting at home indoors and not being able to go out” (Female, breast cancer, 55)

Several participants described feeling as though they had aged more quickly because of inactivity during lockdown and the feeling that the world was “*passing by”* while they were only able to watch.

“one of the things I think about I’m 72 and the thought of old age does depress me. You see things, this railway line will be completed in, I don’t know 2040, oh well I won’t be around to see that, that kind of thing. I don’t enjoy the prospect of getting old. Then Covid on top of it all and being locked up has not helped matters” (Male, prostate cancer, 72)

A small number of people described developing more serious mental health concerns including increased depression, anxiety and anger, which were exacerbated by the restrictions of the pandemic and made daily life much more challenging.

“I’ve had my dark times really being extremely anxious and things go out of control in your head. You know you’re being unreasonable and what you’re thinking is totally out of whack, but almost you feel like you’re in a cage and you can’t get yourself out of it” (Male, prostate cancer, 59)

“A few times over the last year I’ve completely lost it here….just blind anger…I think it was just simmering, simmering, simmering and I forget the reasons why but it just blew and I just lost it…I think it was just all the loneliness and the isolation. You see when there was no pandemic I had the choice to walk around the block a bit.” (Female, breast cancer, 65)

Restrictions on participants’ ability to engage in their regular life often resulted in feelings of frustration, dissatisfaction, boredom and a sense of going *“stir crazy”*. For those cohabiting, these feelings could lead to arguments and tension.

“when you’re confined and there’s only two of you, it doesn’t take a lot for someone to, you know, talk out of turn or just say something that might be irritable to somebody else because you’re confined to the four walls” (Female, colorectal cancer, 62)

For participants living on their own the absence of regular life resulted in feelings of isolation and loneliness. The loss of daily routines and distractions resulted in more time for people to fill, which for some resulted in rumination on past cancer experiences, or anxiety about the future.

Despite the challenges discussed above, many participants found ways to adapt to the restrictions and still fulfil some of the need to make the most out of their life, often finding pleasure in the extra time in which they could explore new activities, interests or connections. Some used the freedom of time to *“take stock”* of the good things in life, plan positive changes going forward and acknowledge their resilience to challenging events.

“we really spent a lot of time focusing on the things that have gone right for us rather than the things that have gone wrong, so it’s been really, really positive.” (Female, colorectal cancer, 58)

“I think emotionally I’ve been quite resilient really. I’ve surprised myself in a way.”

“I always thought I wasn’t very resilient until, since I’ve had cancer, I’ve become more resilient or I’ve noticed that I’m become more resilient than I thought I was”(Female, breast cancer, 40)

It seemed that the challenges of cancer and treatment predisposed some to be more anxious and vulnerable to the uncertainties of the pandemic, with fears of accelerated ageing, sadness about the current restrictions and fear of lost future opportunity punctuating participants’ narratives. Conversely, others appeared to develop some resilience and positive coping mechanisms, with a perspective on life that allowed them to positively adapt to these uncertainties.

## Discussion

The COVID-19 pandemic was a challenge for the United Kingdom and global population, but impacted those LWBC in particular ways. Past cancer experiences influenced the way that the need to feel safe, connected and to make the most out of life were experienced. There are documented increased vulnerability for this group [18] and evidence suggesting people LWBC faced greater challenges in the pandemic [16]. However, participants in this study demonstrated variable awareness of the risks described and a variety of levels of adherence to restrictions. This was exacerbated by confusing guidance, advice from people around them, and clinical or demographic factors such as age, location, comorbidities and enduring symptoms. Despite the variability of risk, those LWBC reported concerns over treatment disruptions, which is consistent with previous research [26, 27]. In keeping with previous literature, the long term effects of cancer and treatment, along with the fear of possible recurrence remained a present concern for many of the participants [11–15]. It was clear that the safety net provided by cancer follow up, no matter how infrequent and routine, remained an important part of the stability of life for those LWBC regardless of how far removed they were from their cancer treatment. This demonstrated the need for more specific and detailed information about adjusted follow up schedules and the important reassurance this would bring.

Like many people during times of crisis [4], people LWBC needed to remain connected to the people and places around them. A mismatch between this need and the reality of their circumstances resulted in many descriptions of boredom, loneliness and isolation. For this population an important moderating factor in the need for connection was the associated need to make the most out of life, having lost time to cancer and treatment. Having already navigated something as challenging as cancer, many participants felt angry that the restrictions enforced by the pandemic stole more time from them, imposing ‘unfair’ additional delays and denial of life they had earned. Losing the healthy future that many had envisaged as a motivator to get through cancer treatment was experienced as a double loss.

In keeping with research prior to the pandemic [9] and research during the pandemic focussing on health behaviours [33], the emotional and social scaffolding people had around them was a key moderating factor in their mental health, whether this was other people or their immediate physical environment, such as having accessible outside spaces. People also appeared to suffer more if they were used to relying on external and social stimulus to regulate their mental health in their everyday lives, such as sports, clubs, employment or regular social engagements. In contrast, those who regulated their mental health from internal resources appeared to be less significantly impacted and more able to adapt to the restrictions and uncertainty.

Further detailed research is needed in this area, but there are implications for future pandemics or health crises resulting in similar social restrictions. The ongoing maintenance needs of people LWBC remain important and can be easily overlooked due to their less immediately active treatment. Simple reassurances and information about adjustments to care plans could have a significant impact on their feelings of safety and stability. Because of the variable perception of risk and applicability of the guidance to them as people LWBC, clearer guidance about risks for them and family are needed. Guidance will need to account for the fact that some people may disregard advice because of wanting to make the most out of their life, or may adhere to the advice strictly, making them vulnerable to isolation. It may be beneficial to develop specific support for this population to help them accurately assess their vulnerability e.g., through mental health instruments such as the highly validated Hospital Anxiety and Depression Scale (HADS) [34], and make practical adjustments accordingly, such as consider the need for pharmacological or psychological interventions. Such interventions could include connecting people LWBC to others in a similar position [4].

Compared to the healthy population, these factors would seem to place people LWBC in a potentially vulnerable position for poorer longer-term mental health outcomes as a result of the pandemic. Longitudinal research is needed to monitor the psychosocial trajectories of this group and further inform interventions, information and support.

### Strengths and limitations

This study gave people LWBC an opportunity to describe their experiences of the pandemic in their own words. The participant-driven approach to the interviews allowed novel areas to be explored and grounded the analysis in experiences that are important to the study population. These novel areas were usually specific examples or stories the participant raised without being prompted by researcher-led questions, which were then explored in detail to allow them time to describe and reflect on these experiences. However, the interviews were primarily focussed on health behaviours and therefore experiences related to psychosocial and mental health needs emerged as a secondary area of interest. In addition it would have been beneficial to discuss with participants what needs were being unmet, and what recommendations for change they had in the event of future global health crises.

### Supplementary Information

**Additional file 1.** ASCOT COVID-19 Interview Schedule V1 21.07.20.

## Data Availability

Details of coding generated during the current study is available from the corresponding author on reasonable request.
