# Supplementary material for: The impact of the COVID-19 pandemic on mental health and quality of life in people living with and beyond breast, prostate and colorectal cancer – a qualitative study
Source: BMC Psychol. 2024 Jan 16;12:25. doi: 10.1186/s40359-023-01471-1 (PMC10790421; doi:10.1186/s40359-023-01471-1)
Supplement: Supplementary file 1 — Additional file 1. ASCOT COVID-19 Interview Schedule V1 21.07.20. [file 40359_2023_1471_MOESM1_ESM.docx]

*NB: The researcher will have access to the participant’s questionnaire answers during the interview. Before they begin they will look over the responses and will therefore know the participant’s employment situation and living arrangements as well as their comorbidities.*

**Semi-structured interview schedule**

*Introduce yourself.*

*Ask permission to record the conversation. Ask them not to use any names so that the recording is anonymous. Then turn recorder on.*

Thank you so much for completing our questionnaire for us. We are collecting some really interesting data. The purpose of today’s call is to allow you to tell us in more detail about how things have been for you this year.

I’ll start with a very general question and then we can perhaps get into more details as we go on if that’s OK. What are the main ways that the coronavirus pandemic has impacted your life?

*Let them answer this however they choose. If they don’t cover the below topics then ask them about them:*

- Did you stay at home during lock-down? Did you go out at all? Have you started going out or having people to visit more now?
- Has the coronavirus pandemic had any impact on your health *(check if they report having had coronavirus before asking this and if so focus on that first) (consider the health conditions they mentioned in the questionnaire and changes to medical care, as well as any reported changes to how they responded to experienced symptoms)?If they say it has then discuss their feelings about this.*
- Has the pandemic resulted in you changing any of your health behaviours, for example your diet or exercise? *Let the participant lead this to start with and discuss all the behaviours they mention having changed. Also look at the answers to qu 51 and discuss any behaviours that they reported change on that they have not spontaneously mentioned.*
- Do you think you will be changing your health behaviours in the coming months?
- Do you feel that your quality of life has improved or worsened over the course of this year? *(consider sleep, loneliness, fatigue, health).*
- Do you think your quality of life will change over the coming months?
- *If they answered anything other than agree and strongly agree to question 57 on the questionnaire (implying that they are not sure or would not be willing to have a COVID-19 vaccination) ask they why they answered as they did and what their reasons are.*
- Is there anything else that you would like to tell us about?
- Do you have any questions?
- *Thank them for their time.*
